# Editorial—Vol. XXIV

**Published:** 1884-08

**Authors:** 


### Editorial.

---

### VOLUME XXIV.

The present number opens the twenty-fourth volume of the JOURNAL. We have to note a change in the editorial management. Dr. Van Peyma, who has borne a full share in the labors and responsibilities of the JOURNAL since it passed from the hands of Dr. Miner, voluntarily retires, thus placing the work entirely in the hands of the two remaining editors. The severance of the editorial relations which have existed during the five years in which we have been associated with our confrere is a matter of deep regret to us, as we are sure it will be to our patrons, who must have appreciated the vigorous honesty and superior ability which he brought to his editorial work. In assuming the labors involved in the management of the JOURNAL, now devolving upon the two editors, we can only give assurance that the JOURNAL will maintain the same independent attitude, upon professional and ethical questions, it has heretofore advocated, not allowing its columns to be the mouthpiece of any clique or school, or the advocate of any party in or out of the profession. The editors, though connected with the Niagara Medical College, and jealous of the interests of a movement which promises so much for the advancement of a higher standard of medical education, do not intend the JOURNAL to be the organ of this or any other institution, but will utter words of "truth and soberness" whenever the interests of the profession here or elsewhere call for fearless expression. We have to reiterate the experience expressed on previous occasions, that this JOURNAL is an exponent of the activity and zeal of the profession. No one pen can infuse earnestness and vigor into its pages, nor incite interest in scientific investigations. We can record the results of labor and diffuse their blessings and fruits to humanity through our wide constituency. Our city has already become an important commercial and medical centre. We will endeavor to be a faithful witness of its progress in scientific and professional subjects. We ask our readers to help us in the important work, which, owing to the retirement of our much respected co-laborator, now devolves upon us.

